# Application of nerve blocks in breast surgery: evolution and prospects from efficacy comparison to multidimensional assessment and precision application

**DOI:** 10.3389/fsurg.2026.1791457

**Published:** 2026-04-29

**Authors:** Cong Deng, Yongxing Xu, Maolin Zhong, Shihong Li

**Affiliations:** 1 The First Clinical Medical College of Gannan Medical University, Ganzhou, China; 2 Department of Anesthesiology, The First Affiliated Hospital of Gannan Medical University, Ganzhou, China; 3 Ganzhou Key Laboratory of Anesthesiology, The First Affiliated Hospital of Gannan Medical University, Ganzhou, China

**Keywords:** breast surgery, erector spinae plane block, nerve block, pectoral nerve block, postoperative analgesia, serratus anterior plane block, thoracic paravertebral block

## Abstract

Postoperative pain represents a significant challenge after breast surgery, emphasizing the need for effective perioperative pain management. Guided by enhanced recovery after surgery (ERAS) principles, multimodal analgesia strategies centered on nerve block have become a prominent clinical research focus. This review comprehensively explores the evolution of nerve block techniques in breast surgery, ranging from the “gold standard” thoracic paravertebral block (TPVB) to newer thoracic fascial plane blocks, including the pectoral nerve blocks (PECS), serratus anterior plane block (SAPB), erector spinae plane block (ESPB), and other innovative approaches. It evaluates the relative benefits and limitations of various techniques regarding analgesic efficacy, opioid-sparing potential, safety, and procedural simplicity. Beyond traditional acute pain management, this review addresses the prevention of chronic post-mastectomy pain syndrome (PMPS), improvements in postoperative recovery quality, and potential effects on oncological outcomes. This review emphasizes the importance of designing individualized precision nerve block strategies based on the surgical scope (e.g., breast-conserving surgery, mastectomy, axillary lymph node dissection, and breast reconstruction) and the distinct features of each technique. No single nerve block technique fits all scenarios; clinical decision-making should focus on selecting the most appropriate approach tailored to the specific patient and procedure. Future research should prioritize high-quality trials for emerging techniques, long-term outcome evaluations, and the development of standardized nerve block protocols to enhance evidence-based practices in this domain.

## 1 Introduction

Breast cancer remains the most prevalent malignancy among women worldwide (1). Surgery remains a cornerstone of comprehensive therapy; however, surgical trauma inevitably introduces a range of perioperative challenges, with postoperative pain being one of the most significant. Up to 60% of patients undergoing breast cancer surgery develop persistent post-surgical pain, with approximately one in four experiencing moderate-to-severe persistent pain (2–4). This not only compromises immediate postoperative comfort and early functional recovery but also serves as a recognized risk factor for the development of chronic postoperative pain syndrome, commonly referred to as post-mastectomy pain syndrome (PMPS) (3, 5). As a result, optimizing perioperative analgesia in breast surgery has become a critical priority for anesthesiologists and surgeons alike.

The introduction of the enhanced recovery after surgery (ERAS) framework has transformed perioperative management in breast surgery (6). Central to these strategies are multimodal analgesia approaches, particularly those centered on nerve block, which have gained significant prominence due to their potent opioid-sparing effects and minimal disruption of physiological functions (7–9). Advances in ultrasound-guided techniques have further enhanced the precision of regional anesthesia, facilitating the development of various thoracic fascial plane blocks, such as the pectoral nerve blocks (PECS), serratus anterior plane block (SAPB), and erector spinae plane block (ESPB). These innovative techniques have increasingly challenged the long-established “gold standard” the thoracic paravertebral block (TPVB) (10, 11).

While numerous comparative studies of nerve block techniques have been conducted, their findings are often inconsistent, and the scope of evaluation has expanded beyond traditional pain scores. This review provides a critical overview of nerve block techniques in breast surgery, moving beyond a simple assessment of the advantages and disadvantages of individual approaches. It provides a comprehensive exploration of their evolution, from traditional methods to innovative techniques, encompassing efficacy comparisons, multidimensional evaluations, and the transition from generalized strategies to precision applications.

This review investigates the neuroanatomical foundations and mechanisms underlying mainstream nerve block techniques, comparing their analgesic efficacy, safety profiles, and operational characteristics. Furthermore, it addresses their role in preventing chronic pain, enhancing quality of recovery (QoR), and potentially influencing oncological outcomes. Finally, the review discusses the development of individualized nerve block strategies tailored to specific surgical procedures and patient characteristics, concluding with an exploration of future research directions.

### 1.1 Literature search strategy

This review is a narrative review that aims to provide a comprehensive and critical overview of the current evidence on nerve block techniques applied in breast surgery. As a narrative review, this work does not follow a formal systematic review protocol and was not registered in a systematic review database. However, to ensure comprehensive coverage of the relevant literature, a structured literature search was conducted using the PubMed and Web of Science databases from inception through December 2025. The search employed combinations of key terms including “breast surgery,” “breast cancer surgery,” “mastectomy,” “nerve block,” “regional anesthesia,” “thoracic paravertebral block,” “pectoral nerve block,” “PECS,” “serratus anterior plane block,” “erector spinae plane block,” “fascial plane block,” “rhomboid intercostal block,” “intertransverse process block,” “postoperative pain,” “chronic pain,” “post mastectomy pain syndrome,” “enhanced recovery,” “quality of recovery,” and “oncological outcomes”. Reference lists of identified articles and relevant reviews were also manually screened to identify additional pertinent studies. Priority was given to randomized controlled trials (RCTs), systematic reviews, meta-analyses, and network meta-analyses published in peer-reviewed journals, while narrative reviews providing important conceptual frameworks were also included where relevant. Non-English publications and conference abstracts without full-text availability were excluded. Article selection was based on relevance and the expert judgment of the authors rather than on predefined systematic eligibility criteria.

## 2 Neuroanatomy related to breast surgery and principles of various block techniques

Effective nerve block for breast surgery requires a thorough understanding of chest wall neuroanatomy. Pain associated with breast surgery arises from multiple sources, including incisional pain, deep tissue pain, and nerve injury, necessitating the development of diverse nerve block techniques targeting specific anatomical sites (12).

### 2.1 Characteristics of sensory nerve distribution in the breast, chest wall, and axillary region

The sensory innervation of the breast and chest wall is primarily supplied by the T2–T6 intercostal nerves, which branch into lateral and anterior cutaneous nerves. The lateral cutaneous branches provide sensation to the lateral chest wall and the outer half of the breast, while the anterior cutaneous branches innervate the anterior chest wall and the inner half of the breast (12).

The intercostobrachial nerve, a lateral branch of T2, supplies sensory innervation to the axillary floor and the inner aspect of the upper arm. Blocking the intercostobrachial nerve is crucial during axillary dissection to mitigate postoperative pain in the axillary region and upper arm (13, 14). Additionally, the upper portion of the breast receives partial sensory input from the supraclavicular nerves (C3-C4) of the cervical plexus. This explains why thoracic nerve block alone may not completely alleviate pain from incisions at the upper pole of the breast (12).

Deep fascial structures of the chest wall are also significant contributors to pain, especially in surgeries involving prosthetic implantation or flap reconstruction. Structures such as the pectoralis muscles (innervated by the medial and lateral pectoral nerves), serratus anterior (innervated by the long thoracic nerve), and latissimus dorsi (innervated by the thoracodorsal nerve) require careful consideration in reconstruction surgeries (15, 16). For a comprehensive visual illustration of the sensory innervation of the breast and chest wall, including the distribution of intercostal nerve branches and the intercostobrachial nerve, readers are referred to the detailed anatomical diagrams provided by Woodworth et al. (12).

### 2.2 Target sites and mechanisms of mainstream nerve block techniques

Nerve block techniques for breast surgery are broadly categorized into four approaches: paravertebral, anterior chest wall, lateral chest wall, and posterior chest wall.

#### 2.2.1 Paravertebral approach: thoracic paravertebral block

TPVB is widely regarded as the “gold standard” for regional analgesia in breast surgery (17). This technique involves the injection of local anesthetic into the thoracic paravertebral space, directly blocking spinal nerves as they traverse the space (18, 19). TPVB provides potent, multi-dermatomal analgesia on the ipsilateral chest wall. However, the proximity of the target space to the pleura and neuraxis necessitates careful risk consideration, which is discussed in detail in Section 3.1 (20, 21). For detailed ultrasound anatomy and needle trajectory during TPVB, readers are referred to the landmark descriptions by Karmakar (22) and the ultrasound-guided technique illustrated by Pace et al. (23).

#### 2.2.2 Anterior chest wall approach: pectoral nerve blocks

PECS is interfascial plane blocks that provide analgesia by injecting local anesthetic into the fascial planes of the chest muscles. PECS I involves injection between the pectoralis major and pectoralis minor muscles, primarily targeting the medial and lateral pectoral nerves (24). PECS II involves deeper injections into the plane between the pectoralis minor and serratus anterior muscles, blocking the lateral cutaneous branches of the intercostal nerves (T2–T6), the intercostobrachial nerve, and the long thoracic nerve (25, 26). Detailed illustrations of the interfascial injection planes for PECS I and PECS II can be found in the original technique descriptions by Blanco (24) and Bashandy et al. (27), which include clear ultrasound-guided anatomical diagrams.

#### 2.2.3 Lateral chest wall approach: serratus anterior plane block

SAPB is another interfascial plane block that can be performed as either a superficial or deep block (28). This technique targets the lateral cutaneous branches of the T2–T9 intercostal nerves through the serratus anterior fascial plane and is particularly effective for breast-conserving surgeries and lateral incisions (29–31). For visual guidance on the SAPB technique, including superficial and deep approaches, readers are directed to the original technique description by Blanco et al. (28) and the procedural illustrations by Mayes et al. (32).

#### 2.2.4 Posterior chest wall approach: erector spinae plane block

The ESPB involves the injection of local anesthetic into the fascial plane between the erector spinae muscle and the transverse process. This technique blocks thoracic and lumbar segmental spinal nerve branches, providing effective perioperative analgesia (33). ESPB has gained widespread use due to its demonstrated analgesic efficacy and favorable safety profile reported in multiple clinical trials (34–36). Notably, the posterior injection site is anatomically distant from critical neurovascular structures and the pleura, contributing to its superior tolerability compared with TPVB, as further examined in Section 3.2.2 (37). The anatomical basis and ultrasound-guided approach of the ESPB are comprehensively described and illustrated in the original report by Forero et al. (33) and the mechanistic review by Chin et al. (38).

Table 1 summarizes the core features of these nerve block techniques, highlighting their key characteristics for clinical consideration.

**Table 1:** Summary of nerve blocks applied in breast surgery.

Parameter	TPVB	PECS (I/II)	SAPB	ESPB
Anatomical Target	Thoracic paravertebral space	Interpectoral plane (I); Pecto-serratus (II)	Superficial or deep to serratus anterior muscle	Deep to erector spinae muscle on transverse process
Primary Nerves Blocked	Spinal nerve roots (ventral and dorsal rami), sympathetic chain	PECS I: Medial and lateral pectoral nerves; PECS II: Additionally lateral cutaneous branches of intercostal nerves (T2–T6), intercostobrachial nerve, long thoracic nerve	Lateral cutaneous branches of intercostal nerves (T2–T9), intercostobrachial nerve, long thoracic and thoracodorsal nerves	Dorsal and ventral rami of thoracic spinal nerves via anterior fascial spread
Dermatomal Coverage	Ipsilateral, typically 4–5 contiguous dermatomes per injection level; multi-level injections can achieve T1–T6 coverage (22)	PECS I: Limited to pectoral motor innervation; PECS II: Anterolateral chest wall (T2–T6) and axillary region (25, 26)	Lateral chest wall (T2–T9), including axillary region; limited coverage of medial/anterior chest wall (10, 28)	Variable and less predictable; potentially T2–T9 depending on volume and spread pattern; dermatomal coverage failure rate ∼55.9% (10, 39)
Estimated Duration of Analgesia (single injection)	12–24 h (22)	12–24 h (10)	8–24 h (10)	12–24 h; variable due to unpredictable spread (10, 37)
Technical Difficulty/Learning Curve	High: Requires precise identification of paravertebral space, pleura, and transverse processes; steep learning curve requiring extensive training (19, 40)	Moderate: PECS I is relatively straightforward; PECS II requires identification of multiple intermuscular fascial planes (10)	Low to Moderate: Clear ultrasound landmarks (rib, latissimus dorsi, serratus anterior); straightforward procedure (10)	Low: Easily identifiable bony landmark (transverse process); short learning curve with high first-attempt success rate for novices (10)
Potential Complications	Pneumothorax, vascular puncture, epidural/intrathecal spread, hypotension, Horner's syndrome (20–22, 41, 42)	Vascular puncture, local anesthetic systemic toxicity, pneumothorax (rare), hematoma (10, 25)	Local anesthetic systemic toxicity (rare), vascular puncture (rare), pneumothorax (very rare due to superficial injection over rib) (10, 43)	Minimal: Mild puncture site pain; no reported cases of pneumothorax, major vascular injury, or neurological injury (34, 37, 44)
Primary Surgical Indications	Major and radical surgeries; all types of breast surgery (17, 20, 21, 41)	Mastectomy, especially with axillary dissection; implant-based reconstruction (10, 25, 45, 46)	Breast-conserving surgery, lateral incisions, procedures involving axillary region (29, 43, 47)	Various breast and thoracic surgeries; particularly useful as a safer alternative to TPVB (34–36)

Duration estimates are based on single-injection technique using long-acting local anesthetics (e.g., 0.25%–0.5% bupivacaine or ropivacaine). Actual duration varies depending on drug selection, concentration, volume, injection technique, and the use of adjuvants such as dexmedetomidine or dexamethasone (48). TPVB, thoracic paravertebral block; PECS, pectoral nerve blocks; SAPB, serratus anterior plane block; ESPB, erector spinae plane block.

## 3 Comparative evidence on nerve block techniques

The evolution of analgesic techniques for breast surgery reflects an ongoing dialogue between traditional techniques, which are considered standards due to their high efficacy but come with technical complexity, and emerging, innovative approaches that prioritize safety and simplicity. The central challenge lies in optimizing analgesia while enhancing safety and accessibility.

### 3.1 Thoracic paravertebral block: efficacy and limitations

Since its introduction in the last century, the TPVB has been regarded as the “gold standard” for regional analgesia in breast surgery due to its robust anatomical basis and well-documented efficacy (7, 17). While numerous RCTs and meta-analyses have reported favorable outcomes for TPVB in terms of postoperative analgesia, opioid reduction, and recovery (49–53), these findings must be interpreted cautiously. Many earlier trials lacked standardized multimodal analgesia in control groups, potentially overstating the incremental benefit attributable to TPVB itself. Furthermore, the considerable variability in injection techniques (single-level vs. multi-level), local anesthetic protocols, and outcome measurement time points limits the comparability of available evidence.

However, despite its exceptional efficacy, TPVB carries significant risks. The procedure targets anatomical structures near the pleura, blood vessels, and nerve roots, making procedural errors potentially catastrophic. Serious complications, such as pneumothorax and vascular injuries, have been reported (20, 21, 41). These challenges limit the broader adoption of TPVB, particularly in non-specialist centers or by less-experienced anesthesiologists. Even with technical advances, residual risks persist, necessitating sound anatomical knowledge and advanced technical skills.

### 3.2 Fascial plane blocks: focusing on enhanced safety and simplicity

In response to the limitations of TPVB, ultrasound-guided fascial plane blocks have emerged as novel and rapidly expanding alternatives. These techniques involve injecting local anesthetics into specific muscle-fascial planes, offering a safer and simpler approach. This innovation has prompted numerous comparative studies evaluating the efficacy of fascial plane blocks relative to TPVB.

#### 3.2.1 Comparison of erector spinae plane block and serratus anterior plane block: comparable analgesia with potential opioid-sparing benefits in erector spinae plane block

A recent meta-analysis and systematic review of RCTs by Shaikh et al. comparing the ESPB and SAPB found no significant differences in postoperative pain scores. However, ESPB was associated with significantly lower opioid consumption within 24 h postoperatively and a longer time to first analgesic request (54).

#### 3.2.2 Comparison of thoracic paravertebral block and erector spinae plane block: analgesic non-inferiority and procedural feasibility

The TPVB remains a widely utilized technique for managing intraoperative and postoperative pain during breast surgery. However, its use is associated with notable risks. In contrast, the ESPB has emerged as a safer alternative that provides comparable perioperative analgesia for breast surgery.

Several RCTs and meta-analyses have directly compared these two techniques, confirming the non-inferiority of ESPB to TPVB in key postoperative analgesic outcomes. For example, Aoyama et al. demonstrated that ESPB fell within the predefined non-inferiority margin for 24 h postoperative fentanyl consumption and the area under the curve for pain scores (55), while Gürkan et al. reported no significant differences in 24 h postoperative morphine consumption between ESPB and TPVB groups (36).

Nevertheless, TPVB exhibits certain advantages in the early postoperative period. Systematic reviews and meta-analyses have revealed that resting pain scores during the first 0–2 h postoperatively were significantly lower in the TPVB group compared to the ESPB group (35, 56). However, by 24 h postoperatively, differences in pain scores and opioid consumption between the two groups are no longer clinically significant (57).

In terms of sensory block coverage and reliability, TPVB outperforms ESPB. A multicenter RCT by Raft et al. found that the dermatomal coverage failure rate was 20.4% for TPVB, compared to 55.9% for ESPB (39). Conversely, ESPB is easier to perform and has a shorter learning curve. Moustafa et al. reported that novice physicians achieved a significantly higher success rate with ESPB compared to TPVB (40). Regarding safety, TPVB carries a higher risk of complications, including pneumothorax, vascular puncture, and total spinal anesthesia (18, 58). To date, serious complications such as pneumothorax or major vascular injury have not been reported in association with ESPB in published clinical trials, although the absence of reported events does not exclude the possibility of rare occurrences. Mild puncture site pain has been reported in fewer than 5% of cases. The favorable safety profile of ESPB is likely attributable to its injection site being located within the superficial fascial layer of the back, at a greater distance from critical structures such as the pleura, paravertebral blood vessels, and the neural axis.

These conflicting data—ESPB's non-inferiority in analgesic outcomes alongside its substantially higher dermatomal coverage failure rate—represent a paradox that the current literature has not fully resolved. One plausible explanation is that adequate clinical analgesia may not require complete dermatomal blockade, particularly when nerve blocks are employed as components of multimodal analgesia. Alternatively, patient self-reported pain scores may lack the sensitivity to detect differences in block coverage that are evident on formal sensory testing. Resolving this discrepancy is critical for understanding the true analgesic mechanism and reliability of ESPB.

#### 3.2.3 Comparison of thoracic paravertebral block with pectoral nerve blocks and serratus anterior plane block: evidence suggesting comparable efficacy with favorable safety profiles

The comparison between TPVB and fascial plane blocks, such as PECS and SAPB, has been extensively studied.

All three techniques provide reliable analgesia, though their effectiveness varies depending on the clinical scenario. According to a meta-analysis by Grape et al., resting pain scores 2 h postoperatively were slightly lower in the PECS II group compared to the TPVB group. However, subgroup analysis revealed no significant differences in pain scores for surgeries involving axillary lymph node dissection (59). Similarly, a systematic review comparing SAPB and TPVB reported no statistically significant difference in resting pain scores at 24 h postoperatively (60). Moreover, a randomized trial comparing SAPB, ESPB, and TPVB found that SAPB and TPVB achieved comparable dynamic pain scores during coughing at 4–8 h postoperatively, with both significantly outperforming ESPB (47).

The relative technical complexity of these approaches also varies considerably (10, 61), a factor that carries important implications for clinical adoption and is discussed further as part of the precision decision-making framework in Section 5.2.

### 3.3 Exploration of emerging techniques: the potential of rhomboid intercostal block and intertransverse process block

Emerging techniques, such as the rhomboid intercostal block (RIB) and intertransverse process block (ITPB), represent ongoing innovation in regional anesthesia for breast surgery. RIB, a posterior chest wall block targeting the intercostal nerves, has demonstrated promising efficacy in breast surgery. Preliminary findings from RCTs and meta-analyses suggest that RIB not only provides effective analgesia but also reduces the incidence of postoperative nausea and vomiting (PONV) (62, 63). Additionally, the ITPB and its modifications, such as the modified thoracic paravertebral block and the modified intercostal block, aim to achieve precise paravertebral diffusion of local anesthetics without the need for direct puncture into the paravertebral space (64). These modifications enhance safety while maintaining analgesic efficacy. Although further large-scale clinical investigations are needed to validate these emerging techniques, their development underscores the field's commitment to advancing safety, precision, and efficacy in regional anesthesia.

### 3.4 Identifying the optimal technique: evidence from network meta-analyses

The conflicting results of comparative studies on nerve block techniques necessitate a broader analytical approach. Network meta-analysis provides a comprehensive perspective by simultaneously comparing and ranking multiple techniques. An et al. conducted a network meta-analysis of 100 RCTs, which highlighted that mainstream regional block techniques such as TPVB, PECS II, SAPB, ESPB, and RIB significantly outperformed the no-block group in terms of analgesia and postoperative outcomes (65). Among these techniques, RIB ranked highest for reducing pain scores in the post-anesthesia care unit, 24-hour morphine consumption, and PONV incidence. However, the authors cautioned that the strength of evidence supporting these findings remains very low. Similarly, network meta-analysis conducted by Singh et al. and De Cassai et al. identified continuous TPVB, SAPB, deep SAPB, and interpectoral-serratus plane blocks as leading options for perioperative analgesia in breast surgery (66, 67).

While the network meta-analysis by An et al. ranked RIB highest across multiple outcomes, these rankings must be interpreted with considerable caution. The authors themselves rated the certainty of evidence as very low, primarily due to within-study risk of bias and imprecision resulting from small sample sizes in the RIB subgroup. Network meta-analysis rankings can be misleading when the underlying evidence is sparse or of low quality, as small changes in effect estimates may substantially alter rankings without reaching statistical or clinical significance. Consequently, these rankings should be viewed as hypothesis-generating rather than definitive clinical guidance.

These varying conclusions reflect significant heterogeneity in study designs, patient populations, and surgical settings. Consequently, rather than aiming to identify a single universally superior technique, clinicians should adopt a patient-specific approach, tailoring regional anesthesia strategies to individual clinical scenarios.

To provide a consolidated overview of the comparative evidence discussed above, Table 2 summarizes the key findings from the major head-to-head comparative studies and meta-analyses on nerve block techniques for breast surgery.

**Table 2:** Summary of key comparative evidence on nerve block techniques in breast surgery.

Comparison	Reference	Study Design	Sample Size	Key Findings	Evidence Quality
ESPB vs. SAPB	Shaikh et al. (54)	Meta-analysis and systematic review of RCTs	550	No significant difference in pain scores; ESPB associated with lower 24 h opioid consumption and longer time to first analgesic request	Low-Moderate
TPVB vs. ESPB	Aoyama et al. (55)	Retrospective propensity-matched noninferiority trial	93	ESPB non-inferior to TPVB in 24 h fentanyl consumption and AUC pain scores	Moderate
	Gürkan et al. (36)	RCT	75	No significant difference in 24 h morphine consumption	Moderate
	Raft et al. (39)	Multicenter RCT	292	Dermatomal coverage failure: TPVB 20.4% vs. ESPB 55.9%	High
	Moustafa et al. (40)	Randomised comparative study	102	Novice physicians achieved higher success rate with ESPB	Moderate
ESPB (vs. control)	Leong et al. (35)	Systematic review and meta-analysis	861	ESPB significantly reduced pain intensity up to 24 h and decreased oral morphine equivalents	Moderate
TPVB vs. PECS	Grape et al. (59)	Systematic review, meta-analysis and trial sequential analysis	388	PECS slightly lower resting pain at 2 h; no difference in axillary dissection subgroup	Moderate
TPVB vs. SAPB	Wang et al. (60)	Systematic review and meta-analysis	1,796	No significant difference in resting pain at 24 h	Low–Moderate
SAPB vs. ESPB vs. TPVB	But et al. (47)	Randomized, prospective, single-blinded study	77	SAPB and TPVB comparable dynamic pain at 4–8 h; both superior to ESPB	Moderate
Network Meta-analysis (global ranking)	An et al. (65)	Network meta-analysis	6,639	RIB ranked highest for PACU pain, 24 h morphine, and PONV; evidence quality very low	Very Low
	Singh et al. (66); De Cassai et al. (67)	Systematic review and network meta-analysis of RCTs	5,686 and 4,074	Continuous TPVB, SAPB, deep SAPB, interpectoral-serratus blocks ranked as leading options	Low-Moderate

Evidence quality ratings are based on the original authors' assessments or on our appraisal of study design, sample size, and risk of bias.

ESPB, erector spinae plane block; SAPB, serratus anterior plane block; TPVB, thoracic paravertebral block; PECS, pectoral nerve blocks; RCT, randomized controlled trial; RIB, rhomboid intercostal block; PACU, post-anesthesia care unit.

## 4 Beyond pain scores: multidimensional evaluation

The evaluation of nerve block techniques in breast surgery has evolved beyond traditional metrics such as pain scores and opioid consumption. A broader, multidimensional framework now emphasizes long-term patient benefits, including the prevention of chronic pain, improvement in the overall quality of recovery, and potential oncological impacts.

### 4.1 Acute pain control and opioid-sparing effects: core benefits and evidence summary

Effective acute pain management and opioid-sparing effects remain the best documented advantages of nerve block techniques. Both TPVB and fascial plane blocks demonstrate significant improvements over no-block controls, as consistently supported by meta-analyses.

Leong et al. conducted a systematic review and meta-analysis, demonstrating that ESPB significantly reduced pain intensity up to 24 h after breast surgery and markedly decreased postoperative oral morphine equivalents (35). Similar benefits have been consistently reported for PECS (8, 68, 69), SAPB (30, 70, 71), and RIB (62, 63), highlighting their role in improving pain control and reducing opioid consumption.

### 4.2 Long-term benefits: hope and controversy in preventing chronic post-mastectomy pain syndrome

Preventing chronic PMPS has emerged as a meaningful metric for evaluating analgesia strategies beyond acute pain management. Regional anesthesia may prevent PMPS by blocking nociceptive input and interrupting central sensitization during and shortly after surgery (72, 73).

Several studies provide encouraging insights. TPVB (74), SAPB (29), and pectoserratus plane block (75) have demonstrated significant reductions in PMPS incidence following breast cancer surgery, with efficacy maintained for up to 6 months postoperatively or longer. Ren et al. emphasized regional anesthesia as a widely used and effective clinical strategy for managing PMPS (76).

However, the evidence remains inconclusive. Studies on TPVB have yielded mixed results. A large double-blind RCT by Albi-Feldzer et al. and a meta-analysis by Harkouk et al. found no significant reduction in chronic pain incidence beyond 3 months, although TPVB may offer preventive benefits for specific subtypes, such as chronic neuropathic pain (77, 78).

Heesen et al. and Elsharkawy et al. highlighted the limitations of current evidence, citing weak and inconsistent findings due to insufficient sample sizes and methodological heterogeneity (79, 80). Elsharkawy et al. further emphasized that high-quality, large-scale, and long-term clinical trials are necessary before nerve block can be widely adopted as a definitive preventive measure for PMPS (80).

The apparent contradiction between positive and negative findings regarding PMPS prevention likely reflects the multifactorial nature of chronic post-surgical pain development. PMPS arises from a complex interplay of surgical nerve injury, perioperative nociceptive input, central sensitization, and psychosocial vulnerability factors. It is plausible that nerve blocks can attenuate one component of this pathway—namely, perioperative nociceptive input, while being insufficient to prevent PMPS when other contributing factors predominate. Future research should stratify patients by risk profiles and examine whether specific subgroups derive greater preventive benefit from regional anesthesia.

### 4.3 Enhancing patient experience: improving postoperative quality of recovery and reducing postoperative nausea and vomiting

Modern anesthesiology increasingly prioritizes patient-centered outcomes, with postoperative quality of recovery (QoR) emerging as a key metric. Multiple studies have demonstrated that nerve blocks significantly improve QoR scores in breast surgery patients.

Abdallah et al. showed that ultrasound-guided multilevel paravertebral blocks combined with total intravenous anesthesia significantly improved QoR scores in patients undergoing outpatient breast tumor resection. These benefits included reduced intraoperative morphine consumption, minimized PONV, and shorter discharge times (81). Similarly, a meta-analysis by Hung et al. confirmed that various peripheral nerve blocks are associated with improved QoR scores, particularly when evaluated using the QoR-40 scale (82).

Nerve blocks also reduce PONV, a common postoperative complication, primarily due to their opioid-sparing effects. By significantly lowering the use of emetogenic opioids such as morphine and fentanyl, nerve blocks eliminate a major trigger for PONV (49, 51–53).

### 4.4 Frontier exploration: the impact of regional anesthesia on tumor recurrence and metastasis—from theory to clinical evidence

The potential impact of regional anesthesia on long-term oncological outcomes remains a promising yet controversial topic. Theoretically, surgical stress, volatile anesthetics, and high-dose opioids suppress immune function, which may promote tumor recurrence and metastasis. In contrast, regional anesthesia may preserve immune function by mitigating stress responses and reducing opioid consumption (83, 84).

Early clinical evidence by Exadaktylos et al. suggested lower recurrence rates with TPVB combined with propofol (6%) compared to general anesthesia with opioids (24%) (85). Deegan et al. further provided molecular-level evidence, showing lower postoperative levels of pro-tumor factors and higher levels of the anti-tumor cytokine IL-10 in patients receiving regional anesthesia (86).

However, more recent studies have cast doubt on these findings. A large retrospective study by Tsigonis et al. using propensity score matching found no significant differences in 5-year overall survival, disease-free survival, or local recurrence rates between regional and general anesthesia groups (87). Similarly, a systematic review by Pérez-González et al., analyzing 15 high-quality studies, concluded that there is insufficient evidence to confirm that regional anesthesia improves breast cancer outcomes (88).

The most definitive evidence to date comes from Sessler et al., who published a multicenter RCT in The Lancet, demonstrating no significant differences in recurrence or survival rates between TPVB with propofol and volatile anesthesia with opioids (89). A 5-year follow-up pragmatic randomized controlled trial by Enlund et al. confirmed no significant divergence in long-term survival curves between propofol and sevoflurane anesthesia groups, solidifying the consensus that clinical evidence supporting oncological benefits of regional anesthesia remains insufficient (90).

The trajectory of evidence in this domain is instructive for the broader field: early observational findings generated plausible biological hypotheses, but subsequent high-quality RCTs failed to confirm a clinically meaningful effect. This underscores the importance of evidence hierarchy and the need for methodological caution when drawing clinical recommendations from retrospective or mechanistic studies alone.

## 5 Toward precision: clinical decision-making

The evolution of analgesic techniques for breast surgery underscores the emergence of precision medicine, which emphasizes individualized assessments as the foundation of contemporary clinical practice. This approach requires clinicians to comprehensively evaluate multiple factors, including surgical scope, technical feasibility, pharmacological considerations, and the anticipated duration of postoperative pain, to determine the optimal nerve block strategy.

### 5.1 Strategy selection based on surgical scope: breast-conserving surgery, mastectomy, axillary dissection, and breast reconstruction

The extent and type of surgery are critical determinants of nerve block selection, as they directly influence the sources of pain and the range of neural innervation involved.

For breast-conserving surgery or excision of small benign tumors, where surgical trauma is primarily confined to the breast tissue and skin, targeted blocks such as PECS or SAPB may be sufficient in many cases, though the decision should be individualized based on anticipated pain intensity and patient-specific factors. These blocks provide localized coverage that aligns with the limited surgical scope. Employing more complex techniques, such as multi-segmental TPVB, may not offer significant advantages over standard multimodal analgesia and could unnecessarily increase procedural time and risk (8, 47, 69, 91). In contrast, mastectomies or modified radical mastectomies require nerve blocks that provide coverage of multiple intercostal nerves, typically from T2 to T6. Techniques such as TPVB, ESPB, or PECS II are commonly employed in these cases. The choice among these options should balance safety, efficacy, and ease of implementation, considerations that are further explored below.

For procedures involving axillary lymph node dissection, the complexity of pain management increases due to the need to address somatic pain from the chest wall as well as the axillary region, which is innervated by branches of the intercostal nerves and the brachial plexus (12). Evidence suggests that PECS II and SAPB, owing to their anatomical coverage, provide more reliable analgesia for the axillary region compared to TPVB or ESPB (26, 31, 92, 93). Therefore, based on the available anatomical and clinical evidence, PECS II or SAPB may be preferentially considered when axillary dissection is planned, although the optimal choice should account for individual patient factors and operator expertise.

Breast reconstruction surgeries present the greatest challenges in nerve block selection. For implant-based reconstruction, particularly subpectoral implant placement, blocks targeting the pectoral nerves, such as PECS blocks, are essential (45, 46). However, for autologous tissue reconstruction using flaps, pain originates from both the recipient site (chest wall) and the donor site (typically the abdominal wall). In such cases, a single chest wall block is inadequate. Atwez et al. introduced the concept of “maximum local regional block” which combines chest wall blocks (e.g., PECS II) with abdominal wall blocks (e.g., Transversus Abdominis Plane Block). This approach has been shown to reduce hospital stays and opioid consumption, offering superior outcomes compared to single-region blocks (94).

### 5.2 Balancing technical feasibility, safety, and the learning curve

After identifying the required neural coverage, clinicians must evaluate available techniques by considering their technical feasibility, safety profile, and associated learning curve. Among the mainstream techniques, SAPB is generally considered to have clear ultrasound landmarks and a relatively straightforward injection process, which may facilitate a shorter learning curve compared to other techniques. PECS requires a two-point injection into anatomically more complex intermuscular planes, representing an intermediate level of difficulty. TPVB demands precise ultrasound identification of the thoracic transverse processes, pleura, and paravertebral space, conferring the highest technical complexity (19, 22). ESPB, despite using a posterior approach, benefits from clear bony landmark visualization and has been shown to be more accessible to novice operators, with Moustafa et al. reporting significantly higher first-attempt success rates compared to TPVB (40). While TPVB provides excellent analgesia, its high technical demands and complication risk limit widespread adoption, particularly in community settings (20, 41). A national survey of Italian anesthesiologists identified “lack of experience/training” as the primary barrier to regional anesthesia adoption (95), underscoring the importance of selecting techniques that balance efficacy with a manageable learning curve.

### 5.3 Optimizing local anesthetic and adjuvant combinations

Precision in nerve block strategies also encompasses pharmacological optimization. Extending the duration of analgesia from a single injection is critical for improving patient satisfaction and reducing the need for postoperative interventions. Adding adjuvants to local anesthetics has emerged as a key strategy in this regard.

A review by Xuan et al. highlighted the utility of two commonly used adjuvants (48). Dexmedetomidine, an α2-adrenergic receptor agonist, enhances block efficacy and prolongs duration by causing local vasoconstriction and slowing the absorption of the anesthetic or among other mechanisms. Dexamethasone, a long-acting corticosteroid, reduces postoperative inflammatory pain and extends the duration of peripheral nerve blocks. These adjuvants improve the effectiveness of single-injection nerve blocks without increasing procedural complexity.

### 5.4 Single injection vs. continuous catheter: matching analgesic duration to patient needs

The final aspect of precision decision-making involves choosing between single-injection and continuous catheter techniques.

Single-injection nerve blocks are preferred for most outpatient procedures or when postoperative pain is expected to last 24–48 h, given their simplicity and efficiency. However, for patients undergoing complex reconstructive surgeries with severe postoperative pain anticipated to persist for several days, single-injection blocks may be insufficient. In such cases, continuous catheter infusion techniques provide a valuable alternative (96). By inserting a fine catheter into the target fascial plane and connecting it to a portable infusion pump, clinicians can achieve prolonged, stable regional analgesia over several days.

Although catheter-based techniques are more complex and require meticulous postoperative management, they are invaluable for carefully selected high-risk patients. Consequently, the decision between single-injection and continuous catheter techniques must balance the patient's pain trajectory with resource allocation and clinical feasibility.

## 6 Discussion

### 6.1 Critical synthesis: comparing techniques across multiple dimensions

When the evidence presented in Sections 3 and 4 is synthesized across multiple dimensions—analgesic efficacy, opioid-sparing effects, safety, technical feasibility, and long-term outcomes—several important patterns emerge that merit critical examination.

Regarding analgesic efficacy, the available evidence demonstrates a convergence rather than a clear hierarchy among mainstream techniques. While TPVB has historically served as the benchmark, direct comparisons reveal that its superiority over fascial plane blocks is largely confined to the immediate postoperative period (0–2 h), with differences in pain scores and opioid consumption becoming clinically negligible by 24 h (35, 56, 57). This temporal pattern has significant practical implications: for outpatient or short-stay breast procedures, the transient early analgesic advantage of TPVB may be offset by its higher complication risk and greater technical demands; conversely, for inpatient procedures requiring extended postoperative analgesia, the demonstrated non-inferiority of ESPB and PECS II becomes particularly compelling.

However, the apparent equivalence in composite pain scores across techniques obscures clinically meaningful differences in other outcome domains. ESPB demonstrates superior opioid-sparing effects compared to SAPB within 24 h postoperatively (54). Available evidence suggests that PECS II and SAPB may provide more reliable axillary coverage compared to TPVB or ESPB in procedures involving axillary lymph node dissection (26, 31, 92, 93), although head-to-head comparisons specifically powered for this outcome remain limited. These nuances are frequently lost in meta-analyses that aggregate heterogeneous surgical populations under uniform outcome metrics.

A critical concern is the inconsistency in sensory block coverage. The substantially higher dermatomal coverage failure rate of ESPB (55.9%) compared to TPVB (20.4%) (39) raises questions about the reliability of ESPB as a sole analgesic strategy for extensive surgeries. This variability likely reflects differences in local anesthetic spread patterns and may be influenced by injection volume, concentration, and individual anatomical variation, factors warranting systematic investigation.

The safety-efficacy trade-off emerges as the most important differentiating factor among techniques. The proximity of TPVB to the pleura and neuraxis confers a fundamentally different risk profile compared to fascial plane blocks, which target superficial muscle-fascial planes distant from critical neurovascular structures (18, 20, 44). When this safety differential is considered alongside the modest and time-limited analgesic advantages of TPVB, the clinical calculus increasingly favors fascial plane blocks for routine breast surgery, particularly in clinical environments with variable operator expertise.

Regarding long-term outcomes, the evidence for PMPS prevention through regional anesthesia remains provocative but inconclusive. The positive findings reported for TPVB (74), SAPB (29), and pectoserratus plane block (75) are counterbalanced by negative results from high-quality trials (77, 78). This discrepancy suggests that the relationship between acute nerve blockade and chronic pain prevention is modulated by factors not yet fully characterized, including the specific pattern of surgical nerve injury, the duration and completeness of perioperative neural blockade, and individual patient susceptibility to central sensitization. Similarly, the definitive negative findings from the multicenter RCT by Sessler et al. (89) and the confirmatory 5-year follow-up by Enlund et al. (90) have largely resolved the debate regarding the impact of anesthetic technique on cancer recurrence, redirecting the field toward more targeted investigations of perioperative immunomodulation.

### 6.2 Quality of evidence and sources of heterogeneity

The inconsistent findings across comparative studies can be attributed to several methodological factors that merit critical scrutiny. First, heterogeneity in baseline analgesic protocols represents a pervasive confound across trials. As noted previously, inadequate control group analgesia may inflate the apparent efficacy of nerve blocks (97). Studies employing rigorous multimodal analgesia in control groups consistently report smaller incremental benefits from nerve blocks, which more accurately reflects contemporary clinical practice.

Second, substantial variability in block execution, including local anesthetic type, concentration, volume, number of injection levels, and adjuvant usage, introduces pharmacological heterogeneity that is rarely controlled for in meta-analyses. For instance, a single-level ESPB with 20 mL of 0.25% bupivacaine cannot be directly compared with a multi-level TPVB using 5 mL of 0.5% ropivacaine per level, yet both may be pooled under their respective technique categories in systematic reviews.

Third, the definition and timing of outcome assessments vary widely across studies. Pain scores measured at rest vs. during movement, at different postoperative time points, and using different rating scales create significant challenges for cross-study comparison. This explains why network meta-analyses conducted by different groups have yielded divergent technique rankings (65–67). The field would benefit from the development and adoption of standardized core outcome sets for breast surgery analgesia research, analogous to those established for other surgical specialties.

These methodological considerations collectively reinforce the view that clinical technique selection should be guided by the totality of evidence incorporating efficacy, safety, technical feasibility, and patient-specific factors, rather than by the results of individual trials or simplified technique rankings.

### 6.3 Future research directions

To address these limitations and advance the field of regional anesthesia, future research should prioritize the following areas:

#### 6.3.1 Designing long-term follow-up studies focused on PMPS prevention

Future studies should prioritize long-term functional outcomes, such as the prevention of PMPS, as primary endpoints. Systematic follow-up is essential to provide definitive evidence on whether regional anesthesia techniques can mitigate chronic pain. Additionally, emerging techniques like RIB and ITPB warrant rigorously designed RCTs to evaluate their efficacy and safety.

#### 6.3.2 Developing standardized multimodal analgesia pathways

Establishing evidence-based multimodal analgesia protocols tailored to specific surgical procedures and patient risk profiles is critical. These pathways should include both primary and alternative regional block options to ensure flexible and comprehensive pain management strategies.

#### 6.3.3 Exploring ultrasound-guided combined block techniques

Complex procedures, such as modified radical mastectomy, require comprehensive perioperative analgesia that often exceeds the capabilities of a single nerve block. For example, the PECS II alone provides insufficient coverage for the medial breast and the nipple-areola complex (12, 98). Additionally, it has limited efficacy in reducing opioid consumption and postoperative pain scores (99).

Combined block techniques, such as the pectoral-intercostal fascial block combined with PECS II, offer promising solutions by achieving broader analgesic coverage and addressing the multidimensional pain pathways involved in complex surgeries (100, 101). These approaches may optimize perioperative outcomes and merit further exploration.

## 7 Conclusion

The field of nerve blocks for breast surgery has undergone a significant paradigm shift, transitioning from reliance on a single “gold standard” technique toward a diversified, evidence-informed approach. This review critically examines this evolution and arrives at several key conclusions.

First, regarding analgesic efficacy, no single nerve block technique demonstrates consistent superiority across all clinical scenarios. TPVB offers robust, multi-dermatomal analgesia with advantages in the early postoperative period, yet its benefits become comparable to fascial plane blocks by 24 h. Based on current evidence, ESPB, PECS II, and SAPB appear to provide clinically comparable analgesia with generally more favorable safety profiles and lower technical barriers, though the quality of evidence varies across comparisons. Emerging techniques such as RIB warrant further investigation given their promising initial results.

Second, from a multidimensional perspective, nerve blocks provide benefits that extend beyond acute pain relief, including opioid-sparing effects and improvements in quality of recovery. However, the evidence for PMPS prevention remains inconclusive, and current data do not support selecting regional anesthesia techniques based on anticipated oncological benefits.

Third, the adoption of a precision approach is essential. Clinical decision-making should integrate surgical scope, anatomical pain pathway considerations, technical feasibility, safety profiles, and patient-specific factors. For breast-conserving surgery, targeted blocks such as PECS or SAPB are appropriate; for mastectomy with axillary dissection, PECS or SAPB should be prioritized for their reliable axillary coverage; and for complex reconstructive procedures, combined block strategies may be necessary.

Finally, advancing the field requires well-designed, large-scale RCTs with standardized multimodal analgesia protocols and long-term follow-up endpoints, rigorous evaluation of emerging techniques, and the development of procedure-specific, evidence-based regional anesthesia pathways. Through the continuing integration of precision medicine principles and patient-centered care, regional anesthesia can consolidate its role as a cornerstone of enhanced recovery protocols in breast surgery.
